# Eluxadoline-induced Recurrent Pancreatitis in a Young Female without a Gallbladder: A Case Report and Literature Review

**DOI:** 10.7759/cureus.3747

**Published:** 2018-12-18

**Authors:** Neelam Khetpal, Lokesh Yadav, Sameen Khalid, Ranjeet Kumar

**Affiliations:** 1 Internal Medicine, Florida Hospital, Orlando, USA; 2 Internal Medicine, Western Michigan University Homer Stryker M.D. School of Medicine, Kalamazoo, USA

**Keywords:** cholecystectomy, eluxadoline, pancreatitis

## Abstract

Eluxadoline is a mixed opioid receptor agonist and antagonist approved for the treatment of diarrhea-predominant irritable bowel syndrome (IBS). It is believed to decrease visceral hypersensitivity without completely inhibiting intestinal motility. Pooled safety data from two phase three randomized trials have reported few cases of pancreatitis especially in patients with sphincter of Oddi (SO) dysfunction and cholecystectomy patients. We present a rare case of eluxadoline-induced recurrent pancreatitis in a 31-year-old female without a gallbladder. Her medical history was significant for irritable bowel syndrome with diarrhea (IBS-D), cholecystectomy, and depression. She was started on 75 mg of eluxadoline (the recommended dose for IBS-D patients without a gallbladder) three weeks prior to the first episode of pancreatitis. She had a recurrent episode of pancreatitis after few weeks and her symptoms and lipase levels improved significantly two days after stopping eluxadoline. The exact mechanism of eluxadoline to cause pancreatitis is unknown but it is believed to increase SO contractions. The absence of gallbladder prevents cholecystokinin mediated relaxation of the SO thus contributing more to spasms with eluxadoline. Few cases of severe pancreatitis and death have been reported even with the reduced dose of eluxadoline recommended for cholecystectomy patients. This case highlights the importance of considering drug-induced pancreatitis and avoidance of eluxadoline even in reduced doses in patients without a gallbladder.

## Introduction

Eluxadoline (Viberzi) is a peripherally acting, mixed opioid receptor agonist and antagonist approved for the treatment of irritable bowel syndrome with diarrhea (IBS-D) [1]. Irritable bowel syndrome (IBS) is the most common functional gastrointestinal (GI) disorder (FGID) characterized by recurrent abdominal pain and altered bowel movements without structural or inflammatory changes. Estimated global prevalence of IBS is 10%-15% [2]. The pathogenesis of IBS is multifactorial and involves visceral hypersensitivity, altered GI motility, alteration of gut microbiota, brain-gut interactions and low-grade inflammation [2]. Most recently updated Rome IV criteria defines IBS as the presence of abdominal pain at least once per week over three months; associated with a change in bowel frequency or consistency and symptom onset should be six months before diagnosis [2].

IBS is further classified into four subtypes based on predominant stool consistency using Bristol Stool Form Scale (BSFS): IBS with diarrhea (IBS-D), IBS with constipation (IBS-C), IBS with mixed stool pattern (IBS-M) and IBS unsubtyped (IBS-U). IBS-D is the most prevalent subtype, constituting 40% of all IBS cases [3]. Multiple treatment options are available for treatment for diarrhea-predominant IBS. Non-pharmacological treatment includes reassurance, education, dietary, and lifestyle changes. Low carbohydrate, low fiber, a diet low in fermentable foods called low fermentable oligosaccharides, disaccharides, monosaccharides and polyols (FODMAP) diet are some of the recommended diets for IBS with variable outcomes. Over the counter (OTC) drugs include probiotics and anti-diarrheal including loperamide. Loperamide is a mu opioid receptor agonist which helps with diarrhea but does not help abdominal pain and bloating and precipitates constipation [3].

Three prescription medications have been approved for the treatment of IBS-D so far. Alosetron is a serotonin receptor agonist which is indicated only for women with severe IBS-D and not responding to other therapies. Rifaximin, a non-systemic antibiotic is another medication the Food and Drug Administration (FDA) approved for IBS-D in May 2015 but the cost may be prohibitive for some patients. Eluxadoline was another drug approved by the FDA in May 2015 for treatment of IBS-D [4]. We present a rare case of eluxadoline-induced recurrent pancreatitis in a female without the gallbladder (Poster presentation: Lokesh Yadav, Tooba Tariq, Sameen Khalid, Ross Driscoll. Eluxadoline Induced Recurrent Pancreatitis in a Young Female without a Gallbladder. World Congress of Gastroenterology at ACG2017 Meeting Abstracts; October 17, 2017).

## Case presentation

A 31-year-old female presented to the emergency department (ED) in January 2017 with complaints of sharp epigastric pain radiating to the back associated with nausea and diarrhea. Past medical history was significant for cholecystectomy in 2010, depression, and IBS-D. She did not have a family history of pancreatitis. She did not consume alcohol or tobacco. Her medications included eluxadoline, bupropion, and oral contraceptive pills. Vital signs included blood pressure 123/78 mm Hg, heart rate 110 beats/minute, sinus tachycardia on telemetry, respiratory rate 18/minute, and oxygen saturation was 100% on room air. On examination, no jaundice or pallor was noted. The patient had epigastric tenderness without rebound. No organomegaly was noted.

She had presented with similar complaints three weeks ago to the ED when she was diagnosed with acute pancreatitis due to epigastric pain and a lipase level of 1067 U/L by Atlanta criteria at that time. There was no abnormality on computed tomography (CT) scan of the abdomen. After stabilization in hospital, she was discharged home with symptomatic management with analgesics, anti-emetics and a plan to advance diet as tolerated. Her symptoms had resolved except for vague abdominal discomfort after meals that was characteristic of her IBS-D.

Further history revealed that the patient was started on eluxadoline 75 mg BID for IBS-D three weeks before the first episode of pancreatitis. This was the reduced dose, recommended for patients without a gallbladder. Lipase level before starting the drug was 31 U/L. During this admission, her lipase level was found to be 1374 U/L. Triglyceride level was 128 mg/dl. Magnetic resonance cholangiopancreatography (MRCP) showed a normal ductal anatomy and absence of stones or tumors. The IgG4 level was normal. Lipase level decreased to 157 U/L within two days of stopping the eluxadoline. Liver function tests and blood counts were normal on both occasions. She was discharged home in a stable condition once her symptoms improved.

## Discussion

Despite IBS being the most common GI disorder, treatment options for IBS-D are inadequate. One of the newer medications for IBS-D recently approved by the FDA is eluxadoline, which acts on mu, kappa, and delta receptor [1]. There are three different types of opioid receptors in the gut, mu (µ), kappa (k) and delta (δ) receptors and all three are G-protein-coupled transmembrane proteins [2]. In the GI tract, these receptor subtypes are present in the enteric nervous system, smooth muscle cells, and interstitial cells of Cajal (ICC) and regulate GI motility, secretion, and visceral sensation. Mu receptor agonists inhibit smooth muscle contractility and thus have the propensity to cause constipation. Delta receptor agonists coexist in same neuronal pathways as mu receptors and exhibit inhibitory effects on smooth muscle [2-3]. Little is known about eluxadoline’s effects on kappa receptors [3].

Eluxadoline is a peripherally acting mu receptor agonist, delta receptor antagonist, and kappa receptor agonist. It is believed to decrease visceral hypersensitivity without completely inhibiting GI motility as compared to selective mu receptor agonists [4]. Mu receptor agonism improves abdominal pain and diarrhea in IBS-D by decreasing GI motility and visceral hypersensitivity whereas delta receptor antagonism prevents mu receptor-mediated unopposed inhibition of GI motility and prevents constipation while enhancing mu receptor mediated analgesia [5-6].

Eluxadoline has a low bioavailability in humans with low systemic absorption and high first-pass metabolism. Eluxadoline is available in 75 mg and 100 mg doses. It is rapidly absorbed in fasting or fed state with maximum concentration decreasing by 50% when administered with high-fat meals. It is recommended to be taken with meals. Mean plasma half-life is 3.5-7 hours. It is mainly excreted in feces (about 80%) and less than 1% is found in urine. Although there is insufficient data showing eluxadoline is cytochrome (CYP) 450 inhibitor, caution is recommended while using strong CYP inhibitors or CYP3A substrates with narrow therapeutic indices such as cyclosporine, tacrolimus, and fentanyl. Studies suggest that eluxadoline could be a substrate for organic anion transporter (OAT) 3, OATP1B1, and multidrug resistance-associated protein 2 and inhibitor of OATP1B1 depending on drug concentrations [7-8].

A randomized phase two, placebo-controlled, clinical trial was conducted by Devo et al. in 807 patients with IBS-D. Doses of 5 mg, 25 mg, 100 mg, and 200 mg twice daily were compared with placebo for twelve weeks. Both 100 mg and 200 mg twice daily showed efficacy in treating IBS-D but due to the increased side effects with 200 mg, 100 mg twice daily was preferred [9].

Lambo et al. conducted two (IBS 3001 and IBS 3002) multicenter, phase three randomized, double-blind, placebo-controlled trials to assess the safety and efficacy of eluxadoline in IBS-D patients [1]. Approximately 2427 patients meeting criteria for IBS-D using Rome III criteria were assigned to either eluxadoline (75 mg or 100 mg) or placebo twice daily. Both studies involved double-blind treatment for 26 weeks to evaluate the efficacy. Additional twenty-six weeks of double blinded treatment was included in the IBS-3001 trial to assess long-term safety followed by two weeks follow-up after the drug was discontinued. The primary endpoint is a composite decrease in abdominal pain and stool frequency on the same day for more than 50% days through weeks 1-12 (FDA endpoint) and weeks 1-26 European Medicine Agency (EMA endpoint). Inclusion and exclusion criteria are shown in Table 1.

**Table 1:** Inclusion and exclusion criteria Inclusion and exclusion criteria for IBS-3001 and IBS-3002 trials to assess the safety and efficacy of eluxadoline [1]. IBS-D: irritable bowel syndrome with diarrhea.

INCLUSION CRITERIA
An average score for worst abdominal pain 3 or more on a scale of 0-10
Average stool consistency was ≥5 on the Bristol Stool Form Scale (BSFS) for at least 5 days. (0=hard lumps; 7= watery diarrhea)
Average IBS-D global symptom score for symptoms of IBS-D 2 or more
EXCLUSION CRITERIA
Inflammatory bowel disease
Previous history of pancreatitis
Alcohol abuse or binge drinking
Sphincter of Oddi dysfunction (SOD)
Post-cholecystectomy biliary pain and cholecystitis within past six months

During the initial twelve weeks, patients in eluxado­line 75 or 100 mg groups had a significantly greater response in the primary endpoint compared to placebo in both studies. From weeks 1 through 26, only the 100-mg arm performed sig­nificantly better than placebo in the primary endpoint in IBS-3001. Both 75 mg and 100 mg groups performed significantly better than placebo in the IBS-3002 trial through week 26 [1,6].

Eluxadoline was first approved by the FDA in May 2015 for treatment for IBS-D. From May 2015 through February 2017, 120 cases of severe pancreatitis or death were reported to the FDA through FDA Adverse Event Reporting System (FAERS) database [10]. Serious cases of pancreatitis or death occurred only after one to two doses of eluxadoline in 48 patients. Fifty-six out of sixty-eight patients with reported gallbladder status had no gallbladder and majority were taking the recommended dosage of eluxadoline. A total of 67 patients were hospitalized and two patients died. One was associated with pancreatitis and one with sphincter of Oddi spasm (SOS). Gallbladder was absent in both of them. In the light of above findings, FDA issued a drug safety communication on March 3rd, 2017 and contraindicated the use of eluxadoline in patients without a gallbladder due to risks of severe pancreatitis and death [2,10].

In a pooled safety data from phase two and two phase three trials, most common adverse events were nausea, constipation, and abdominal pain. A total of six cases met the Atlanta criteria for pancreatitis, defined as having at least two of the following three findings [4]:

1. Epigastric pain radiating to the back with an onset of such pain considered the onset of pancreatitis 

2. Elevation of amylase or lipase three times the upper limits of normal

3. Characteristic findings on imaging 

One case (in the 100 mg group) was consistent with SOS. Three out of remaining five cases were associated with heavy alcohol use, one had biliary sludge and one patient had discontinued eluxadoline two weeks prior to the event hence deemed unrelated to study drug. None of the pancreatitis cases involved multi-organ failure or local or systemic complications [1,2,4]. A total of ten events consistent with SOS occurred in the eluxadoline group (0.8% in 100 mg and 0.2% in 75 mg group). Eight out these ten cases presented with abdominal pain and elevation of liver enzymes; one occurred as pancreatitis and one had abdominal pain with mild elevation of lipase. All of these occurred in patients without a gallbladder. Majority of these cases occurred in the 100 mg group, within two weeks of initiation of therapy and resolved within one week of discontinuation [2,4]. Based on these findings, the lower dose of 75 mg twice daily was recommended for patients without a gallbladder and caution was advised to monitor for symptoms of abdominal pain, elevation of liver enzymes, and pancreatitis. In addition, 75 mg twice daily was also recommended in patients unable to tolerate 100 mg dose, those with mild to moderate hepatic impairment and those concomitantly using OATP1B1 inhibitors [11]. It was contraindicated in patients with known or suspected biliary duct obstruction or sphincter of Oddi dysfunction (SOD), alcoholism and drinking more than three alcoholic beverages/day since alcohol is known to alter periductal anatomy and increase sphincter of Oddi pressure, history of pancreatitis or known structural pancreatic disorder and severe hepatic dysfunction (Child-Pugh class C) [6,11].

SO is a smooth muscle at the confluence of the common bile duct and pancreatic duct that controls the flow of digestive juices through the ampulla of Vater. It helps in regulating the flow of bile and pancreatic secretions into the duodenum [12]. Cholecystokinin (CCK) release following ingestion of fatty meal causes contraction of the gallbladder, relaxes SO, reduces SO tonic pressure and inhibits SO phasic contractions. The exact underlying mechanism of eluxadoline causing SOS and pancreatitis in patients without a gallbladder remains unclear; however, studies have shown that cholecystectomy is associated with suppression of cholecystokinin (CCK) mediated inhibition of SO phasic activity despite normal bile duct pressure and SO motility. It is likely due to damage of nerves connecting gallbladder and SO during cholecystectomy. Elevation of SO basal pressure increased retrograde phasic contractions and increase in phasic wave more than seven contractions per minute (tachyoddia) are manometric changes associated with SOD in cholecystectomy patients [12-13]. Opioids exert a direct effect on SO and are believed to cause SOS and pancreatitis. Mu agonist activity of eluxadoline might be responsible for increased frequency and amplitude of SO while non-mu receptor agonism results in increased basal pressures. These effects are further augmented in cholecystectomy patients due to lack of response to CCK mediated inhibition of SO phasic activity and absence of gallbladder as a pressure reservoir for increased ductal pressures [2,12]. Drug-induced pancreatitis constitutes 0.1%-2% cases of acute pancreatitis but should be considered in the differential diagnosis of pancreatitis especially in the absence of other classical risk factors such as gallstones and alcoholism. Any potentially offending drug should be immediately withdrawn and if pancreatitis improves, the suspicion of drug-induced pancreatitis further increases [14].

## Conclusions

Based on the efficacy data from one phase two and two phase three clinical trials, eluxadoline is an effective drug approved for the treatment for IBS-D. IBS-D is initially managed with lifestyle modifications and OTC medications. Among the prescription medications, rifaximin and eluxadoline should be used before alosetron. It is a logical choice in patients who can not get rifaximin or do not respond to initial treatment. Eluxadoline has generally been well tolerated with rare events of SOS and severe pancreatitis in patients without a gallbladder and excessive alcohol use. In light of the severe adverse events, including death found during post-marketing surveillance, eluxadoline should not be used in patients without a gallbladder even at the previously recommended lower doses; risks and benefits should be evaluated carefully in the context of the severity of symptoms in patients being prescribed with eluxadoline.
